# Uncommon mucosal metastases to the stomach

**DOI:** 10.1186/1477-7819-7-62

**Published:** 2009-08-03

**Authors:** R Kanthan, K Sharanowski, JL Senger, J Fesser, R Chibbar, SC Kanthan

**Affiliations:** 1 Department of Pathology & Laboratory Medicine, College of Medicine, Saskatoon, Saskatchewan, Canada; 2 Department of General Surgery, College of Medicine, Saskatoon, Saskatchewan, Canada

## Abstract

**Background:**

Metastases to the stomach from an extra-gastric neoplasm are an unusual event, identified in less than 2% of cancer patients at autopsy. The stomach may be involved by hematogenous spread from a distant primary (most commonly breast, melanoma or lung), or by contiguous spread from an adjacent malignancy, such as the pancreas, esophagus and gallbladder. These latter sites may also involve the stomach via lymphatic or haematogenous spread. We present three cases of secondary gastric malignancy.

**Methods/Results:**

The first is a 19-year-old male who received a diagnosis of testicular choriocarcinoma in September 2004. Metastatic malignancy was demonstrated in the stomach after partial gastrectomy was performed to control gastric hemorrhage.

The second is a 75-year-old male, generally well, who was diagnosed with adenocarcinoma of the lung in September 2005. Poorly differentiated adenocarcinoma of the lung was demonstrated in a subsequent biopsy of "gastric polyps".

The third is an 85-year-old man with no known history of malignancy who presented for evaluation of iron deficiency anemia by endoscopy in February 2006. Biopsies of the colonic and gastric mucosa demonstrated moderately differentiated invasive colonic adenocarcinoma with metastatic deposits in the stomach.

**Conclusion:**

While the accurate recognition of these lesions at endoscopy is fraught with difficulty, pathological awareness of such uncommon metastases in the gastric mucosa is essential for accurate diagnosis and optimal patient management.

## Background

Primary gastric cancer is the second highest cause of global cancer mortality accounting for over 700,000 deaths annually [1]. Gastric cancer is curable if it is detected early; however many patients are diagnosed with late stage disease wherein despite advances in management protocols, current therapeutic strategies still remain far from optimal [2,3]. Current strategies including surgery and combination chemotherapies provide modest survival benefits in advanced gastric cancer resulting in an overall 5 year survival rate less than 24% [4,5].

Secondary gastric cancer however is a rare event and remains a challenging clinical problem [6,7]. The most commonly described primary sites are breast, melanoma and lung [8,9]. The recognition of such metastases to the stomach outside of findings at autopsy is rare [10]. Higgins found 64 cases of metastatic carcinoma in the stomach among 31,541 autopsied cases while Davis and Zollinger reported 67 metastatic tumours in the stomach among 23,109 autopsied cases [11,12]. However, with enhanced overall survival of these patients these lesions are also being diagnosed with the increased use of esophagogastroduodenoscopy [13].

The clinical presentation of upper gastrointestinal bleeding as a manifestation of gastric metastases is unusual. Further, gastrointestinal bleeding due to secondary gastric choriocarcinoma is uncommon [14]. Metastases to the stomach from a testicular germ cell tumour is extremely rare though autopsy findings indicate a much higher incidence [15]. We will now share our experience of three uncommon metastases to the stomach recognized by histopathological examination of mucosal biopsies obtained at upper gastroendoscopy.

## Case Reports

### Case No. 1

A 19 year old male presented in September 2004 with a two month history of a left testicular mass. This individual was in good health, a non-smoker, and had no significant medical history. Left inguinal orchidectomy yielded a diagnosis of predominantly choriocarcinoma with a small focus of embryonal carcinoma. The lesion appeared confined to the testis with no apparent vascular or lymphatic invasion. A few weeks later the patient complained of blurry vision, fatigue and headache. Multiple brain metastases were identified by CT scans. Detailed imaging confirmed the presence of bilateral metastases to the lungs. He was aggressively treated with chemotherapy. One month later, the patient presented with melena and falling haemoglobin levels. Gastroscopy revealed mild pangastritis and evidence of prior gastric bleeding, with no obvious ulceration or masses. Shortly thereafter uncontrolled gastric hemorrhage necessitated a partial gastrectomy. Metastatic testicular choriocarcinoma was confirmed on histopathological examination of the resected stomach.

#### Histopathology

**Figure 1:**
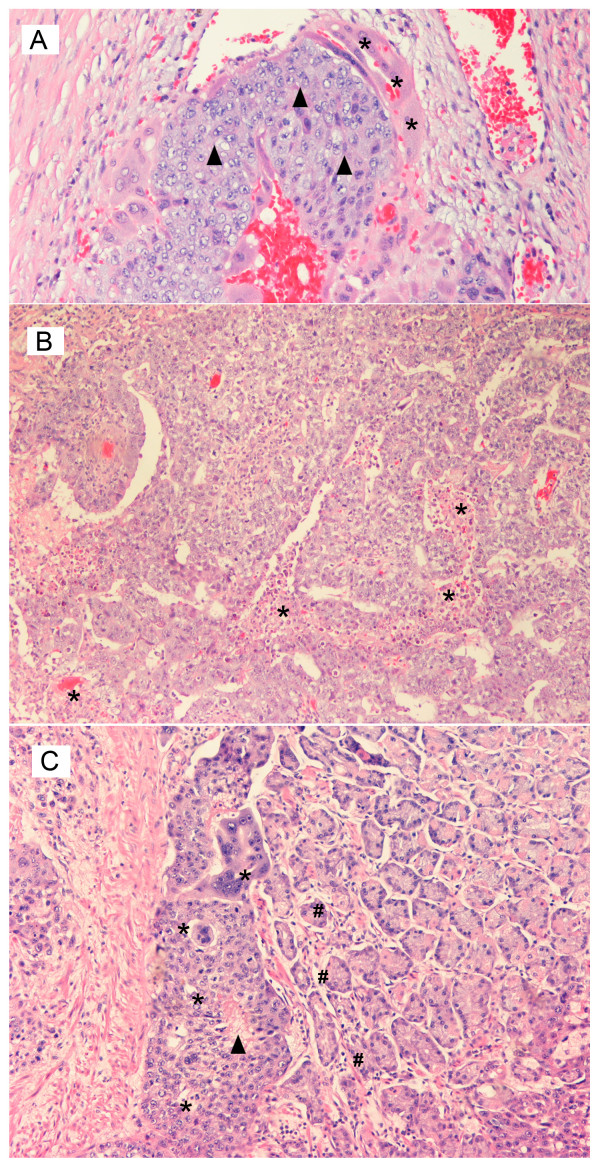
**Histopathology of the Testis Hematoxylin and eosin stained, medium power, magnification ×250**. A. Mixed germ cell tumor of the testis – highlighting a focus of primary choriocarcinoma as seen by the presence of black triangle – cytotrophoblast and * – syncytiotrophoblast cells. B. Mixed germ cell tumor of the testis – highlighting a focus of embryonal carcinoma associated with * – multiple foci of necrosis. C. Metastatic testicular choriocarcinoma – as seen by the presence of * – metastatic trophoblastic cells with areas of black triangle – central necrosis admixed with # – gastric glands.

Figure 1A illustrates the primary choriocarcinoma of the testicle as characterized by a biphasic proliferation of malignant trophoblastic cells: centrally, cytotrophoblastic cells (black triangle) which have clear cytoplasm and mild to moderate nuclear pleomorphism, and above these a "cap" of syncytiotrophoblast cells (*), which demonstrate abundant amphophilic cytoplasm, smudged nuclear chromatin and multinucleation. Often, hemorrhage and necrosis are seen centrally within the mass of cytotrophoblasts. If syncytiotrophoblast cells are inconspicuous, this lesion may be difficult to differentiate from embryonal carcinoma with cellular degeneration.

Figure 1B demonstrates the tubular pattern of embryonal carcinoma component identified in the primary testicular cancer as illustrated with multiple foci of necrosis (*). The cells have basophilic cytoplasm with indistinct borders with large nuclei. The nuclear membranes are irregular with coarsely clumped chromatin with one or more prominent nucleoli. Mitoses and apoptotic bodies are easily demonstrated.

Figure 1C confirms the presence of metastatic choriocarcinoma with its characteristic biphasic population of trophoblastic cells (*) that are easily identified adjacent to the benign gastric glands (#). This focus of choriocarcinoma also has central necrosis (black triangle) within the cytotrophoblasts.

### Case No. 2

An 85-year-old male presented in February 2006 with a diagnosis of iron deficiency anemia. This individual had a history of hypertension, and had recently suffered a small lacunar infarct, but there was no known history of malignancy. On pan-endoscopic examination, a lesion was noted in the right colon at the junction of the cecum and ileocecal valve and on the lesser curvature of the stomach. Biopsy confirmed these lesions to represent moderately differentiated colonic adenocarcinoma, and colonic adenocarcinoma metastatic to the stomach, respectively.

#### Histopathology

**Figure 2:**
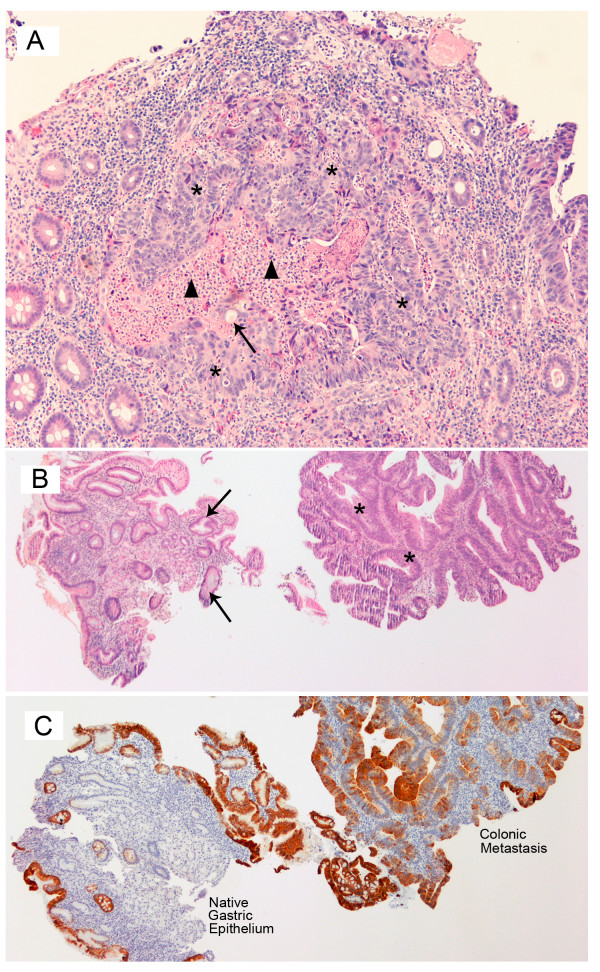
**Histopathology of the Endoscopic Colonic Biopsy Hematoxylin and eosin stained, medium power, magnification ×250**. A. Adenocarcinoma of the colon demonstrated by * – individual nests of "garlandlike" masses with ↑ – punched out lumens and black triangle – central necrotic debris. B. Metastatic colonic adenocarcinoma in the lesser curvature of the stomach as seen by * – malignant epithelium recapitulating colonic glands associated with ↑ – benign gastric glands. C. Metastatic colonic adenocarcinoma in the lesser curvature of the stomach confirmed by immunohistochemical staining of CK20 positive colonic epithelium with negative staining in the gastric epithelium.

Figure 2A represents the colonic biopsies that confirm the presence of a moderately well-differentiated adenocarcinoma of the colon. Well-formed glands with cribriform architecture lined by cuboidal to low columnar epithelium are seen as individual nests or "garlandlike" masses (*). Punched-out lumens (↑) and central necrotic debris (black triangle) are frequently identified. The individual cells demonstrate some retention of polarity, moderate nuclear pleomorphism, prominent nucleoli and frequent mitotic figures.

Figure 2B represents the endoscopic gastric mucosal biopsies with the presence of benign gastric glands, (↑) adjacent to malignant epithelium recapitulating colonic glands (*). The benign gastric epithelium were negative for both CK7 and CK20 (figure 2C), while the malignant colonic epithelium was positive for CK20 (figure 2C) and negative for CK7 supporting the dual population of cells in the gastric mucosal biopsies.

### Case No. 3

A 75 year-old male presented in September 2005 with a two-month history of weight loss. He had also noticed increasing breathlessness and cough over the past month and a half. He was generally well, took no routine prescription medication, and was a non-smoker. There was no known history of malignancy. On plain X-ray, a right sided lung lesion was noted, which proved to have an appearance suspicious for bronchogenic carcinoma on subsequent CT. Adenocarcinoma was confirmed by fine needle aspirate of the lesion and a pleural biopsy. As the patient also complained of significant epigastric and right upper quadrant pain, gastroscopy and biopsy of "gastric polyps" was undertaken. Dysplastic cells most in keeping with poorly differentiated metastatic adenocarcinoma of the lung were identified within the gastric lymphatics.

#### Histopathology

**Figure 3:**
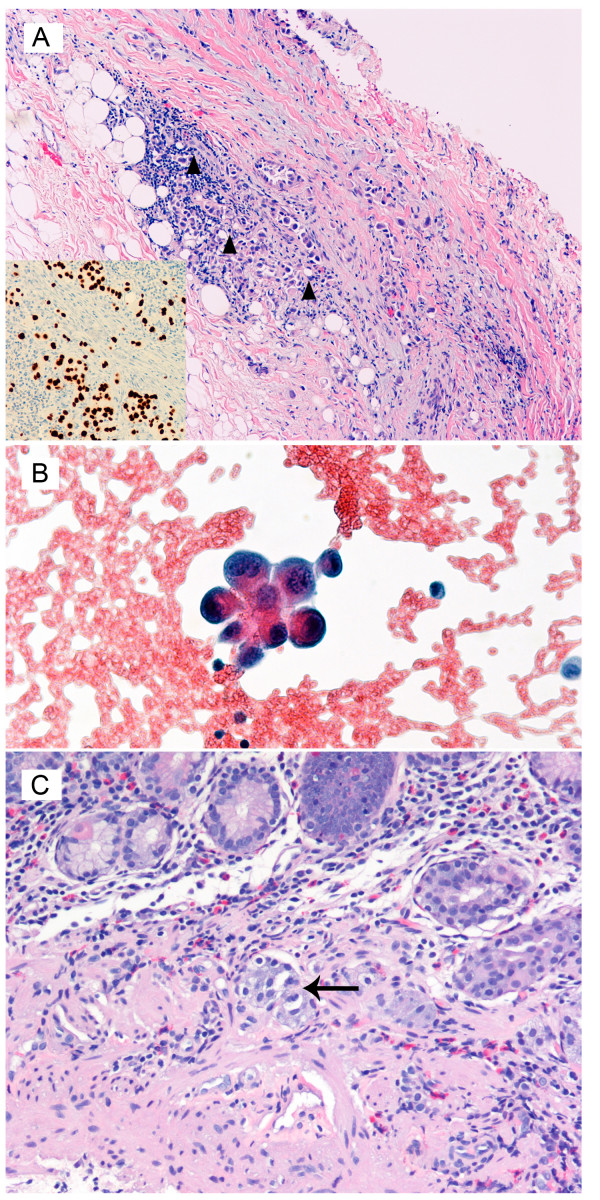
**Histopathology of the Pleural Biopsy Hematoxylin and eosin stained, low power magnification ×150**. A. Pleural biopsy confirming the presence of black triangle – atypical neoplastic cells infiltrating the fibro connective tissue and adipose tissue of the pleura. The inset in the bottom left shows positive immunohistochemical staining with TTF1 supporting primary lung carcinoma. B. Pleural fluid demonstrates the presence of atypical cells with a high nucleus cytoplasmic ratio supporting a neoplastic lesion. C. Mucosal biopsy of the stomach showing the presence of large atypical malignant cells in the vascular channels with ↑ – enlarged hyperchromatic pleomorphic nuclei consistent with poorly differentiated carcinoma from the lung.

A right-sided pleural biopsy as seen in figure 3A illustrates the fibroconnective and adipose tissue of the pleura infiltrated by atypical cells (black triangle) with ample cytoplasm, large, hyperchromatic nuclei, irregular nuclear membranes and prominent nucleoli. The cells have ample cytoplasm, some of which appear to have vacuoles with prominent desmoplasia. Immunohistochemically, these cells were positive for pankeratin, TTF-1 (shown as insert), Ber-EP4 and CEA; they were negative for calretinin, cytokeratin 5 and 6, S100 and Melan-A. The cytological examination of the pleural fluid (figure 3B) demonstrates atypical cells with a high nuclear-to-cytoplasmic ratio suspicious for an underlying malignant neoplasm.

Pathological examination of the endoscopic biopsy of the gastric polyp (figure 3C) shows the presence of large cells with enlarged, hyperchromatic, pleomorphic nuclei (↑). These cells bear resemblance to those identified in the biopsy of the pleura, and reflect poorly differentiated adenocarcinoma with loss of nuclear polarity, increased nuclear size, hyperchromasia, and abundant mitotic figures consistent with metastases from the lung. [The grading of adenocarcinoma (into poorly-, moderately- and well-differentiated) is based on the degree and extent of glandular formation. Poorly-differentiated lesions have few abortive or poorly formed glands, or may grow as sheets of tumour cells].

## Discussion

The involvement of the stomach by metastases is rare with the most common reported primaries include melanoma, and carcinomas of the breast and lung [7,16]. The estimated incidence of gastric metastases at autopsy in individuals with a known malignancy varies from 1.7% [17] to 5.4% [Oda [10]]. Up to half of individuals harbouring such metastases are symptomatic, most commonly with bleeding, pain, vomiting and anorexia [18].

Choriocarcinomas of the testis account for only 0.3% of testicular tumours [19], and gastric involvement by primary testicular germ cell tumour is extremely rare [14,15,20]. Aydiner et al claim the first report of choriocarcinoma metastatic to the stomach in 1993.[21] Testicular choriocarcinoma is most commonly identified in patients in their second and third decades presenting with varied symptoms including hemoptysis, lumbar back pain, GI bleeding, neurological symptoms and endocrinological abnormalities [22]. Choriocarcinomas in particular have a marked affinity for angioinvasion, and disseminate rapidly, disproportionately frequently to the brain [15]. As would be expected from the clinical presentation, it is estimated that half of the patients with testicular germ cell tumours will have metastases at diagnosis, with the most common destinations being, in addition to the brain, the lymph nodes, liver, and lung [14]. Metastases are relatively uncommon to the GI tract, spleen and adrenals [17]. Typically, these lesions respond promptly to chemotherapeutics, and for this reason it is essential to identify metastases to the GI tract, and surgically resect these if feasible. Occasionally, the therapeutic response of the lesion to chemotherapy may result in haemorrhage or intestinal perforation [23]. As primary gastric choriocarcinoma has also been described, an additional diagnostic conundrum for the pathologist involves evaluation of whether the gastric mucosal biopsy represents a primary or a 'true' secondary lesion from an occult testicular primary. While the initial presentation, often dramatic, will draw clinical attention to the metastasis rather than the primary, the accurate diagnosis hinges upon awareness that the gastric lesion could be metastatic, and proceed with a full clinicopathological evaluation to include/exclude a silent testicular primary.

Adenocarcinoma of the lung is now the most frequent form of lung carcinoma in the United States [24], most frequently seen in non-smokers and females. This tends to be a peripherally located lesion, and consequently patients demonstrate late symptoms related to the increasing size of the tumour, distant metastases, and invasion of the pleural compartment. Lymphatic and hematogenous metastases are common, and adenocarcinoma of the lung has the highest incidence of intrapulmonary metastasis [25]. Likewise, adenocarcinoma is the most frequent form of lung carcinoma observed to yield gastric metastases [9,26]. As observed with testicular primaries, acute upper gastrointestinal bleeding and gastric perforation secondary to metastatic lung adenocarcinoma following systemic chemotherapy have been reported [27,28]. It is essential not to forget the possibility of metastatic disease in the differential diagnosis of the patient presenting with a gastric lesion [29] This is further complicated by the fact that adenocarcinoma metastatic to the lung may be virtually indistinguishable from primary gastric adenocarcinoma at histopathological evaluation. Kim et al reported a case of metastatic poorly differentiated carcinoma which, radiologically and endoscopically, was featured like polypoid primary gastric carcinoma [7]. Further to compound the problem, the primary lung adenocarcinoma may not be readily apparent, as in Yamamoto et al's report of lung adenocarcinoma metastatic to the stomach, in which the 4-cm primary tumour in the left lung was not detectable on plain X-ray [30]

Colonic adenocarcinoma is the second most common cause of cancer mortality in North America [31]. Lymphatic and hematogeous metastases to the liver typically occur once the submucosa has been invaded by the way of the portal venous system. While 20% of patients have distant metastases at diagnosis, gastric metastases resultant from colon cancer has been reported infrequently [9,10].

The appearance of metastases to the stomach at endoscopy is variable. The appearance on imaging or gross inspection is generally not suggestive of the primary. Gastric involvement may be characterized by a single lesion in the gastric body or by multiple lesions [6,10]. Often the lesion is described as a "volcano-like" ulcer [18]. The metastases may have the clinical appearance of a primary stromal gastric tumour [19]. Since metastases to the stomach can present before the primary malignancy declares itself, there is danger of mistaking these metastases for primary gastric cancer, and consequently failing to recognize the true primary. Of particular interest to the pathologist is that adenocarcinomas of gastrointestinal or pancreatobiliary origin may adhere to the gastric glands and pits, preserving this morphology even as pits themselves are destroyed mimicking an in-situ 'pseudoprimary' gastric lesion. Similarly, malignancy metastatic from the breast may illicit marked desmoplasia within the stomach so as to convincingly simulate linitis plastica [19].

Gastric metastases may be recognizable as abnormalities on gastroscopy; however as the morphology is variable there are no characteristic appearances that define metastatic disease [10]. Likewise, the appearance on CT scans of metastatic neoplasms to the stomach are indistinguishable from that of gastric primary malignancies, such as adenocarcinoma or lymphoma, and can also be easily confused with the appearance of food residue or inadequate gastric distension. On barium X-ray, these lesions often are described as "target lesions" with the lesion itself depicted as a filling defect and a central collection of barium within it, likely related to the ulcerated morphology seen endoscopically. Frequently, bridging mucosal folds are noted which suggest a submucosal mass. Unfortunately, this "bulls' eye" lesion is also in keeping with a multitude of neoplastic and non-neoplastic conditions of the stomach, including lymphoma, carcinoid, Kaposi's sarcoma and gastric ulcers [32].

In conclusion as pitfalls abound in the clinical presentation, diagnostic imaging and histopathology, it is essential to be acutely aware of both common and uncommon metastases to the stomach and to appropriately include these in the differential diagnosis of all gastric lesions for accurate diagnosis and optimal patient management.

## Consent

Written consent for research and publication was obtained from the patient or their relative.

The consent forms are mailed to the editorial staff.

## Competing interests

The authors declare that they have no competing interests.

## Authors' contributions

RK is the corresponding, and first author of this manuscript. KS and JLS are undergraduate students who have contributed to the acquisition of data, analysis, and interpretation of data. JF is the postgraduate student who presented these three cases in part as a poster presentation at the Canadian Association of Pathologists' Annual Meeting in July 2006. RC and SCK have made substantial contributions to the conception and design of this manuscript. All authors read and approved the final manuscript.
